# Efficacy and practice of facemask use in general population: a systematic review and meta-analysis

**DOI:** 10.1038/s41398-022-01814-3

**Published:** 2022-02-01

**Authors:** Hui Li, Kai Yuan, Yan-Kun Sun, Yong-Bo Zheng, Ying-Ying Xu, Si-Zhen Su, Yu-Xin Zhang, Yi Zhong, Yi-Jie Wang, Shan-Shan Tian, Yi-Miao Gong, Teng-Teng Fan, Xiao Lin, Nina Gobat, Samuel Yeung Shan Wong, Emily Ying Yang Chan, Wei Yan, Si-Wei Sun, Mao-Sheng Ran, Yan-Ping Bao, Jie Shi, Lin Lu

**Affiliations:** 1 Peking University Sixth Hospital, Peking University Institute of Mental Health, NHC Key Laboratory of Mental Health (Peking University), National Clinical Research Center for Mental Disorders (Peking University Sixth Hospital), Chinese Academy of Medical Sciences Research Unit (No.2018RU006), Peking University, 100191 Beijing, China; 2 National Institute on Drug Dependence and Beijing Key Laboratory of Drug Dependence, Peking University, 100191 Beijing, China; 3 School of Public Health, Peking University, 100191 Beijing, China; 4 Peking-Tsinghua Centre for Life Sciences and PKU-IDG/McGovern Institute for Brain Research, Peking University, Beijing, China; 5 Nuffield Dept of Primary Care Health Sciences, University of Oxford, Oxford, UK; 6 JC School of Public Health and Primary Care, The Chinese University of Hong Kong, Hong Kong Special Administrative Region, China; 7 Collaborating Centre for Oxford University and CUHK for Disaster and Medical Humanitarian Response, School of Public Health and Primary Care, Prince of Wales Hospital, Sha tin, Hong Kong; 8 Department of Social Work and Social Administration, University of Hong Kong, Hong Kong, China

**Keywords:** Scientific community, Psychiatric disorders

## Abstract

In recent decades, respiratory infections, including SARS, HINI and the currently spreading COVID-19, caused by various viruses such as influenza and coronavirus have seriously threatened human health. It has generated inconsistent recommendations on the mandatory use of facemasks across countries on a population level due to insufficient evidence on the efficacy of facemask use among the general population. This meta-analysis aimed to explore (1) the efficacy of facemask use on preventing respiratory infections, and (2) the perceptions, intentions, and practice about facemask use among the general population worldwide. We searched PubMed, MEDLINE, Web of Science, Cochrane, bioRxiv, and medRxiv databases since inception to August 17, 2020. From 21,341 records identified, eight RCTs on facemask in preventing infections and 78 studies on perception, intention, and practice of facemask use among the general population were included in the analysis. The meta-analysis of RCTs found a significant protective effect of facemask intervention (OR = 0.84; 95% CI = 0.71–0.99; *I*^*2*^ = 0%). This protective effect was even more pronounced when the intervention duration was more than two weeks (OR = 0.76; 95% CI = 0.66–0.88; *I*^*2*^ = 0%). The meta-analysis of observational studies on perception, intention, and practice on facemask use showed that 71% of respondents perceived facemasks to be effective for infection prevention, 68% of respondents would wear facemasks, and 54% of respondents wore facemasks for preventing respiratory infections. Differences in perception, intention, and practice behavior of facemask use in different regions may be related to the impact of respiratory infections, regional culture, and policies. The governments and relevant organizations should make effort to reduce the barriers in the use of facemasks.

## Introduction

In recent decades, respiratory infections, including SARS, HINI and the currently spreading COVID-19, caused by various viruses such as influenza and coronavirus have seriously threatened human health, of which the novel virus with the capacity to efficiently spread with sustained human-to-human transmission may trigger a pandemic [1]. Medical facemasks (or surgical masks) are routinely used as personal protective equipment to protect people from influenza and other respiratory infections in healthcare settings, by providing a physical barrier against potentially infectious droplets [2]. Although a recent meta-analysis suggested that wearing facemasks could significantly reduce the risk of virus infection [3], particularly for airborne diseases, there has been heated debate continuing during the initial stage of COVID-19 pandemic on the effectiveness of facemask use by the general public in the community settings to prevent the transmission. It has generated inconsistent recommendations on the mandatory use of facemasks across countries at a population level due to insufficient evidence on the efficacy of facemask use among the general population [4]. As with overtime changes, WHO, as well as other national disease control departments like US CDC, have finally recommended that masks should be used as part of a comprehensive strategy of measures to suppress transmission of COVID-19 [5].

The efficacy of facemask use on preventing respiratory infections is still controversial, especially in community with insufficient proofs. Several randomized controlled trials (RCTs) on the efficacy of facemasks have been conducted in community settings, including households [6–10], university residence halls [11, 12], and Hajj Pilgrims tents [13], while, given many of the studies were just conducted over a single season and low adherence of facemask use, they are still not able to provide conclusive results. Previous studies including two meta-analyses on the efficacy of facemask use for preventing transmission of pandemic influenza [14, 15] also provided inconsistent conclusions, while a recent meta-analysis including 14 randomized controlled trials did not support a substantial effect on transmission of laboratory-confirmed influenza. [14] A meta-analysis suggested that disposable surgical masks or reusable 12–16-layer cotton masks were associated with protection from viral transmission in non-healthcare setting. [3] However, it did not differentiate the evidence of surgical masks and general cotton masks, especially in the non-health care setting including multiple settings of community, household, and family contacts. [3] Recent two studies (a rapid review on COVID-19 and a meta-analysis) demonstrated that facemask use could reduce the risk of respiratory infections transmission [16, 17]. However, these studies included some of the clinical trials using the hand sanitizer and facemask as intervention instead of only facemask, which might overestimate the efficacy of facemask use. Thus, more convincing evidence of the efficacy of wearing facemasks in general population is urgently needed.

Beside the efficacy, the perception, intention, and practice towards facemasks use in general population is vital for the adherence of facemask use during pandemics or epidemics. The attitude towards facemasks use among the population should be crucial for predicting the results of the related policies. Evidence shows that the self-reported perception, intention, and practice towards facemask use in the general population vary in different countries or regions [18, 19]. According to the health belief model, perceived susceptibility, severity, barriers, benefits, and cues to action have an influence on the practice of facemask-wearing [20, 21]. However, there is no integrated evidence on the perception, intention, and practice towards facemasks use during pandemics including COVID-19 from a global view, which will support the governments and disease control departments to make evidence-based recommendations on facemask use in the control of respiratory infections and to reduce morbidity and mortality associated with the pandemic among general population worldwide.

Therefore, this systematic review and meta-analysis aimed to firstly evaluate the efficacy of medical masks use on reducing the respiratory infection in community settings, and secondly estimate the perception, intention, and practice regarding wearing facemasks among the general population during infectious disease pandemic.

## Methods

### Search strategy and selection criteria

This systematic review and meta-analysis followed the Preferred reporting items for systematic reviews and meta-analyses (PRISMA) guidelines (Table S1). We included RCTs exploring the efficacy of wearing facemask on reducing respiratory infection in community settings, in which study subjects were assigned into intervention and control groups using random allocation. Second, we included observational studies to evaluate the perception, intention, and practice regarding wearing facemasks among general populations. We searched PubMed, MEDLINE, Web of Science, Cochrane, medRxiv, and bioRxiv databases since inception to August 17, 2020, with no language restrictions. The search terms were developed in collaboration with a research librarian (See Appendix). All articles were double screened (by Hui Li and 1 Ying-Ying Xu) on title and abstract. All full-text articles identified were reviewed by Yong-Bo Zheng, Si-Zhen Su, and Yu-Xin Zhang. Two independent reviewers (Hui Li and Si-Zhen Su) extracted data from included studies.

The studies on the efficacy of facemasks use were included if they met the following criteria: (1) concerning the relationship between medical masks and preventing respiratory infection in community settings (an open setting without confinement and special care for the participants); (2) applying RCT design; and (3) providing complete data of cases and controls for calculating an odds ratios (ORs), relative risks (RRs) with 95% confidence interval (CI). The exclusive criteria were as follows: (1) the subjects were health care workers or studies conducted in hospital-specific settings; (2) the intervention was not using facemasks alone (e.g. combining the facemasks and hand hygiene); and (3) reviews, guidelines, theoretical models and non-research based communications such as letters to the editor.

The studies reporting perception, intention, and practice towards facemask use in general population were included. The inclusion criteria were: (1) concerning the perceived efficacy of facemask for preventing respiratory infection, the intention to wear facemasks or the practice of facemask use in the past; (2) the studies among the general population; and (3) providing complete data for prevalence calculation. Exclusive criteria were as follows: (1) the included subjects were health care workers; (2) no certain data given to obtain the prevalence; and (3) reviews, guidelines, theoretical models and non-research-based communications such as letters to the editor. We registered our systematic review on PROSPERO (number: CRD42020191447).

### Assessment of study quality

Two authors (Yong-Bo Zheng and Yi Zhong) assessed the quality of all included studies. The quality of RCTs was assessed in accordance with Cochrane Handbook for Systematic Reviews of Interventions. The Joanna Briggs Institute (JBI) critical appraisal checklist was used for observational studies. Disagreements were resolved by a third author (Si-Zhen Su).

### Data extraction

The data were independently extracted from eligible papers by researchers (Li H, Zheng YB, Zhong Y and Wang YJ) and the extracted data were subsequently cross-checked. Discrepancies were discussed until a consensus was reached. The following information was extracted from each study of RCTs: (1) first author, (2) year of publication, (3) facemask using places, (4) research site (country), (5) total sample size, (6) intervention designs, (7) follow up times, (8) influenza-like illness (ILI) case definition, and (9) the results of the risk for ILI infection after the interventions (see Table 1). The following information was extracted from each of cross-sectional studies: (1) first author, (2) year of publication, (3) total sample size, (4) type of epidemics of infectious diseases, (5) research site (country), (6) age of participants, (7) perceived efficacy, (8) intervention designs, and (9) practice about facemask use (see Table 2).

**Table 1:** Characteristics of the RCTs investigating the efficacy of facemasks.

Study	Place	Country	Total sampling	Interventions	Follow up	ILI case definition	Results
Aiello et al. [1]	University residence halls	USA	1437 university hall residents (1297 analyzed)	Facemasks, facemasks + hand hygiene, control	6 weeks	Cough and at least 1 constitutional symptom [fever/feverishness, chills, or body aches]	• No statistically significant difference in all arms • Significant reductions in ILI during weeks 4–6 in the facemask + hand hygiene arm
Aiello et al. [12]	Residence halls	USA	1178 university hall residents (1111 analyzed)	Facemasks, facemasks + hand hygiene, control	6 weeks	Cough and at least 1 constitutional symptom [fever/feverishness, chills, or body aches]	• No statistically significant findings in all arms • Significant reductions in ILI during weeks 3–6 in the facemask + hand hygiene arm, with a maximum reduction of 75% during the final study week
Barasheed et al. [13]	Pilgrims tents	Australia	Index = 75; contacts = 89	Supervised facemask tent, no supervised facemask tent	5 days	Fever plus one respiratory symptom [dry or productive cough, runny nose, sore throat, or shortness of breath]	• Significantly less contacts with ILI in the facemask arm than in the control arm
Cowling et al. [6]	Households	HK, China	Index = 162; contacts = 266	Facemasks, hand hygiene, control	9 days	Definition 1 = fever ≥ 38 °C or at least 2 symptoms [headache, coryza, sore throat, aches or pains in muscles or joints, cough, or fatigue]; Definition 2 = at least 2 signs and symptoms [fever ≥ 37.8 °C, cough, headache, sore throat, aches or pains in muscles or joints]; Definition 3 = fever≥37.8 °C plus cough or sore throat	• No statistically significant difference in the facemasks arm or hand hygiene arm
MacIntyre et al. [9]	Households	Australia	Index child = 84; contacts = 218	Surgical masks, P2 masks, control	14 days	Fever > 37.8 °C, feeling feverish or a history of fever, >2 symptoms (sore throat, cough, sneezing, runny nose, nasal congestion, headache), or 1 of the symptoms listed plus laboratory confirmation of respiratory viral infection	• No statistically significant difference in all arms • Adherent use of P2 or surgical masks significantly reduces the risk for ILI infection
Suess et al. [8]	Households	Germany	Index=84; contacts=218	Facemasks, facemasks + hand hygiene, control	8 days	Fever plus cough or sore throat	• No statistically significant difference in two intervention arms in intention to treat analysis • Significantly lower risk of influenza if the pooled data from two intervention arms was implemented within 36 hours after symptom onset of the index patients
Canini et al. [7]	Households	France	Index=105; contacts=306	Facemasks (used by index case), control	7 days	Fever > 37.8 °C or at least 2 symptoms [sore throat, cough, runny nose, or fatigue]	• No statistically significant difference in two arms
MacIntyre et al. [10]	Households	Beijing, China	Index=245; contacts=597	Facemasks (used by index case), control	7 days	Fever ≥ 38 °C plus one respiratory symptom including cough, nasal congestion, runny nose, sore throat or sneezes	• No statistically significant difference in two arms

**Table 2:** Characteristics of included cross-sectional studies.

Study	Participants	Disease	Region	Age	Perceived efficacy	Intention	Practice
Abdulah et al. [86]	1343	COVID-19	Iraqi Kurdistan	16–95	–	–	69.2%
Agüero et al. [75]	1627	H1N1	Spain	18+	–	–	8.4% wore facemask at least once
Ahmad et al. [57]	60 university students	Respiratory infection	Pakistan	Mean age of above 21	–	–	45%
Akan et al. [22]	402 first year university students at Yeditepe University	H1N1	Turkey	–	32.4% very effective; 32.9% moderately effective	–	–
Al-Jasser et al. [76]	1507 pilgrims	2009 Hajj	Saudi Arabia	21–83	–	–	56.5%
Allison et al. [58]	503 students	Influenza	USA	–	–	97% would use masks in a pandemic	30% of students wore masks in week 1, while 15% wore masks in week 2
Al-Mohrej et al. [59]	1149	MERS	Saudi Arabia	11+	–	–	10.9% wore masks in public places
Alqahtani et al. [60]	25 pilgrims	2014 Hajj	13 countries	21–61	–	–	Day 1 45.2%, Day 2 51.8%, Day 3 60%, Day 4 76%, Day 5 60%, Day 6 52%, Day 7 68%
Alqahtani et al. [61]	150 pilgrims	2014 Hajj	Australia	–	18+	75% very effective	–
Alqahtani et al. [62]	344 pilgrims	2017 Hajj	Saudi Arabia	16–79	–	–	53%
Alzoubi et al. [68]	592 university Students	COVID-19	Jordan	–	68.4% of the participants believed that facemask can prevent viral transmission	–	64.7% wore facemask
Ayhan Baser, D., et al. [87]	1070	COVID-19	Turkey	19–83	–	–	39.3%
Azlan et al. [69]	4850	COVID-19	Malaysia	–	–	–	51.2% wore a facemask when going out in public
Azman et al. [63]	30 pilgrims	2013 Hajj	Malaysia	–	–	–	53.33 % wore a facemask when necessary; 23.33% worn a facemask in crowded places
Balaban et al. [64]	186 USA travelers	2009 Hajj	USA	–	–	–	48.90%
Barr et al. [65]	2081 adults completed the module	Influenza	Australia	16+	–	59.9% willing to wear a mask if pandemic influenza were to occur	
Beckage et al. [94]	1004	COVID-19	US	all ages	–	75.5%	
Bowman et al. [95]	3431 complete responses (HK:1663; UK:1768)	COVID-19	HK, UK	18+	–	–	HK:98.8%; UK:3.1%
CDC. [28]	2231	Influenza	USA	18–97	–	82.4% would wear a mask while waiting at the doctor’s office or hospital if asked to by their healthcare provider	–
Chan et al. [66]	1020	H7N9	Hong Kong, China	15+	94.4% thought it was useful for prevention	–	39.0% always or usually worn mask when sick
Chaudhary et al. [67]	400 students of class 9th to 12th	H1N1	India	–	97% of the students perceived use of mask as most effective way to prevent them from swine flu	89.7% willing to facemask when the students were asked about the method, they will use to protect them if they have to visit the patient of swine flu	40% worn masks to protect them from getting infected with swine flu
Chuang et al. [29]	1745	Influenza	Taiwan	20+	–	91.63% Intention to wear a facemask should there be an influenza pandemic	–
Chen et al. [70]	8569 primary school students	COVID-19	China	6–13	–	–	51.60% had a good behavior of mask-wearing
Cheng et al. [96]	10050	COVID-19	Hong Kong, China	–	–	–	96.6%
Chen et al. [88]	2887	COVID-19	China, Japan, South Korea, Western Europe (ie, England, France, Germany, Spain, and Italy), and the US	–	–	–	99.4% in mainland China, 38.7% in Japan, 85.5% in South Korea, 1.6% in Western Europe, 2.1% in the US
Clements [25]	1034	–	USA	18+	–	–	23.6% reported wearing a mask when leaving home
Cowling et al. [71]	Survey 1, Jan 20–23 (*n* = 1,008) Survey 2, Feb 11–14 (*n* = 1,000) Survey 3, March 10–13 (*n* = 1,005)	COVID-19	Hong Kong, China	All ages	–	–	Survey 1-3: 4·5%; 97·5%; 98·8%
Deris et al. [30]	394 pilgrims	2007 Hajj	Malaysia	50.4 ± 11.0	–	–	72.9% wore facemasks during the Hajj
Etingen et al. [31]	3113	H1N1	USA	22-95	–	–	17.15%
Ferdous et al. [72]	2017	–	Bangladesh	12–64	–	–	98.7% of the participants wore a facemask in the crowded place
Gautret et al. [32]	274 pilgrims	2009 Hajj	France	23–83	–	–	40.9% frequently worn a surgical facemask
Geldsetzer et al. [27]	2986 USA + 2988 UK	COVID-19	USA + UK	18+	37.8% of US + 29.7% of UK thought that wearing a common surgical mask was “highly effective” in protecting them from acquiring COVID-19	–	–
Griffiths et al. [33]	359 students	H1N1	Hong Kong, China	–	–	–	47.9% worn a facemask in crowded places
Gu et al. [34]	825 university students	H1N1	China	–	–	9.3% would wear facemasks if they had influenza-like symptoms	–
Gunasekaran et al. [73]	1697	–	Malaysia	–	–	–	99.70%
Hashim et al. [35]	468 pilgrims	2013 Hajj	Makkah and Malaysia	17–84	–	–	72% of pilgrims used surgical facemasks and N95 facemasks
Haischer et al. [93]	5517 shoppers entering retail stores	COVID-19	US	–	–	–	41.5% of the observed sample wore a mask
Hayat et al. [74]	1257	COVID-19	Pakistan	16–49	The participants (81.3%) believed that wearing a mask could help in the prevention of COVID-19	–	wore a mask when they moved out of their homes (85.8%)
Hezima et al. [89]	812	COVID-19	Sudanese	18+	–	–	34.1%
Hickey et al. [23]	773 migrant participants	H1N1	Thailand	–	33% Believes that using a facemask could prevent transmission of illness	12% Would agree to wear facemask after exposure to someone who is sick; 8% Would agree to wear facemask while waiting at a health facility	25% had used facemasks in the past when sick
Huang et al. [77]	10,198	COVID-19	China	–	–	–	97.9% (*n* = 9986) used masks in public
Ikpama et al. [25]	1086	COVID-19	Nigeria	20+	–	–	59.1% usually employ the use of facemasks
Jang et al. [36]	1005	MERS	Korea	19+	–	–	15%
Kamate et al. [37]	791	H1N1	India	18+	Facemasks (36.6%) were rated as one of the most effective methods for the prevention of Influenza A (H1N1)	–	–
Kantor and Kantor [90]	1005	COVID-19	US	18+	–	–	Performed in last week: Always 7.1%, Most of the time 4.0%, Sometimes 4.0%
Lau et al. [38]	1397	SARS	Hong Kong, China	18–60	81.70%	95%	64%
Lau et al. [39]	820 travelers returning to Hong Kong by air	SARS	Hong Kong, China	15–60	–	–	75.7% of the respondents wore a mask all the time or most of the time during the flight; 15% of the respondents reported wearing a mask in public areas at the visited destination most or all of the time
Lau et al. [18]	863	SARS	Hong Kong, China	18–60	92.7% believed that using a mask in public places is efficacious means of SARS prevention	71.2% of all the respondents would wear a mask in public places	–
Lau et al. [40]	1603	SARS	Hong Kong, China	18–60	–	–	74.3% reported a high frequency (i.e. frequently or very frequently) of facemask-wearing
Lau et al. [41]	503	H5N1	Hong Kong, China	18–60	90.5% perceived High/very efficacy of prevention measures	73.8% willing to wear facemask in public venues; 92.4% willing to wear facemask in public venues when having ILI symptoms	–
Lau et al. [42]	302	H5N1	Hong Kong, China	18–60	92.1% perceived high/very high efficacy for prevention of bird-to-human H5N1 infection		36.6% Often/always wearing facemask in public venue when suffering from ILI in the last 3 months
Lau et al. [19]	550	H1N1	Hong Kong, China	18–60	93.3% perceived a facemask as an efficacious measure to control the spread of the virus (quite or very efficacious)	90%	13.5% reported ever having worn facemasks in public venues
Lau et al. [43]	999	H1N1	Hong Kong, China	18+	Very effective 24.0%; Quite effective 69.0%	–	21.5% worn facemasks regularly in public areas; 88.7% worn facemasks when going out in case of ILI symptoms
Lee et al. [79]	973	COVID-19	South Korea	18	wearing facial masks (*M* = 3.72, SD = 0.49)	–	63.2% reported always wearing a facial mask when outside
Liu et al. [80]	608	COVID-19	China	All ages	–	–	83.70%
Matusiak et al. [91]	2307	COVID-19	Poland	18–27	–	–	60.4%
Meilicke et al. [44]	4012	H1N1	Germany	18+	–	2009, 36.4%; 2008, 32.6%	–
Memish et al. [45]	432 pilgrims	2009 Hajj + H1N1	Saudi Arabia	13–94	44.7% Wearing a mask is a way to avoid H1N1 infection	–	35.10%
Mo et al. [46]	300	H1N1	Hong Kong, China	18–60	–	89.33%	–
Mohammed et al. [47]	457 pilgrims	2016 Hajj	Saudi Arabia	14–80	–	–	60.40%
Rahman and Sathi [92]	441	COVID-19	Bangladesh	18+	–	–	91.4% Wearing masks when going outside the home
Szepietowski et al. [82]	2307 students	COVID-19	*Poland*	18–27	–	–	60.4% had used facemasks during the previous week
Quaife et al. [81]	213	–	Kenya	18+	–	–	94% of respondents reported “always” wearing a mask outside of their house
Tang et al. [48]	1002	SARS	Hong Kong, China	18+	–	–	61.20%
Tang et al. [78]	1329	SARS	Hong Kong, China	19+	–	–	61.2% of the respondents reported consistent wearing of facemasks to prevent contracting and spreading SARS
Taylor et al. [49]	2081	Influenza	Australia	16+	–	58% would be very/extremely willing to wear a facemask	–
Taylor et al. [50]	2007 (2081 participants) + 2010 (2038 participants)	H1N1	Australia	16+	–	56.9% willing to wear a facemask	–
Tobaiqy et al. [83]	1012 pilgrims	2019 Hajj	48 nationalities	–	–		34.6% pilgrims always used facial masks in crowded areas
Uchida et al. [51]	11,390 children	Influenza	Japan	Grade 1–6	–	–	52.00%
Van Cauteren et al. [52]	10,076	H1N1	France	0–14, 65+	–	–	11.3% of the cases older than 14 years used a facemask when they were sick
Wada et al. [53]	3129	Influenza		20–69	–	–	15.2% frequently worn a facemask in public
Wadood et al. [84]	305 university students	Bangladesh	17–28	–	–		53.8% reported wearing surgical facemask when out in public
Wong et al. [54]	230 adolescents	SARS	Hong Kong, China	–	–	–	47.8% of respondents indicated consistent practice
Wu et al. [55]	13,003	Influenza	China	18+	–	–	20.9% reported using facemask when going to hospitals
Xu et al. [85]	8158	–	China	18+	–	–	97.9% reported wearing facemask
Zhang et al. [56]	7121	Influenza	China	18+	–	–	55.9% use of hospital masks
Zhong et al. [24]	6910	COVID-19	China	–	–	–	98.0% wore masks when going out

### Data analysis

For the data from RCT studies concerning the relationship between facemask use and preventing respiratory infection in community settings, we used inverse variance weighted random effect models to pool the log-transformed odds ratios (ORs) and relative risk (RRs) from primary studies. If multiple models were presented within a study, we selected the multivariable model in each study for meta-analysis. Heterogeneity across studies was measured using the *I*^*2*^ statistics with the chi-square *p* value. Subgroup analysis was used to explore the relationship between facemask use and risk of respiratory infections in different duration, intervention designs and settings.

The pooled prevalence of the attitudes and behaviors towards facemasks was estimated by inverse variance weighting random-effects modeling. Subgroup analysis was conducted on the basis of different diseases, broad WHO regional classification, facemask use places and situations for study-specific effect estimates. Meta-regression was also used to assess the differences between these subgroups.

Sensitivity analyses were performed to assess the influence of each study, omitting the studies with the largest weight on the overall result one by one. Publication bias was investigated using funnel plots and formally tested using Begg’s test and Egger’s tests. All of the statistical analysis was performed using STATA 12 software, and values of *p* < 0.05 were considered statistically significant.

## Results

**Fig. 1:**
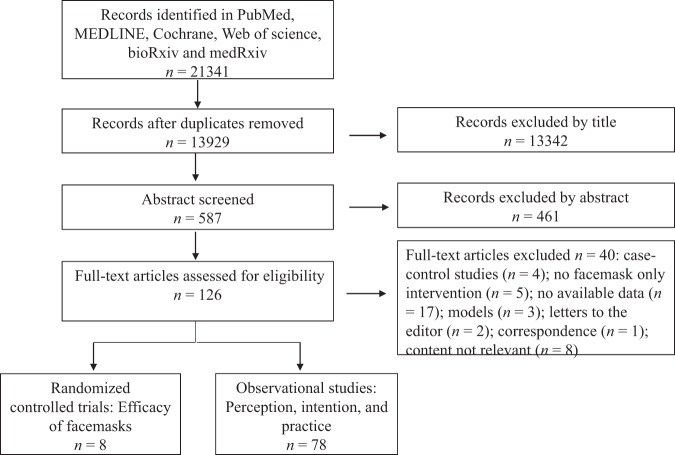
Summary of the literature search and inclusion process.

We identified 21,341 articles in the initial database search, of which 126 were retrieved based on their titles and abstract content. After excluding 40 articles that did not meet our inclusion criteria (see Table S2 for exclusion reasons), eight were eligible for RCT studies on the efficacy of facemask use, and 78 were eligible for studies about perception, intention, and practice towards wearing facemasks based on our inclusion criteria (see Fig. 1).

### Efficacy of facemask use

Characteristics of the eight RCT studies investigating the efficacy of facemasks are presented in Table 1. A total of 5,242 participants were included. Included RCT studies on estimating the efficacy of facemasks had been conducted in different settings. Five of these studies were conducted within households [6–10]. Two studies from the same group focused on the impact of facemasks on the incidence of ILI infection in university residence halls [11, 12]. A pilot RCT tested the efficacy of facemask use in the tents among Australian Hajj Pilgrim [13]. Among the studies conducted in households, three required both the index and the contacts or only contacts to wear facemasks, while two estimated the efficacy of facemasks as source control [7, 10]. Two studies were conducted with follow up more than two weeks [11, 12], while other six studies were followed up in a range of 5–14 days.

**Fig. 2:**
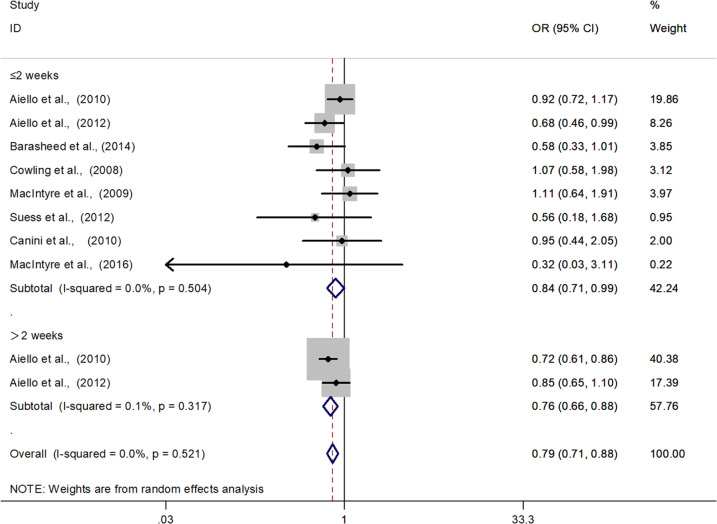
Forest plots of meta-analysis to determine efficacy of facemasks in reducing respiratory infection among the general population. There is a significant protective effect if the duration of facemask use was more than two weeks. The results are expressed as odds ratios (ORs) and 95% confidence intervals (95% CI).

Meta-analysis of eight studies showed a significant protective effect (Fig. 2. ≤ 2 weeks, *N* = 5242; OR = 0.84; 95% CI: 0.71–0.99; *I*^*2*^ = 0%). In the university residence halls, this protective effect was more pronounced if the intervention duration was more than two weeks (Fig. 2. > 2 weeks, *N* = 2261; OR = 0.76; 95% CI: 0.66–0.88; *I*^*2*^ = 0%). The subgroup analysis of intervention settings (households, resident halls or tents) and population (by index, contacts or both contacts and index) did not show any significant difference (Fig. S1, S2).

### Perception, intention, and practice towards facemask use

Characteristics of the 78 studies [18, 19, 22–96] investigating the perception, intention, and practice of facemask are presented in Table 2. A total number of 151,228 participants were included, with 14 studies reported (14,556) the rates of perception, 15 studies (17,651) reported the rates of intention, and 63 studies (151,228) reported the rates of practice.

**Fig. 3:**
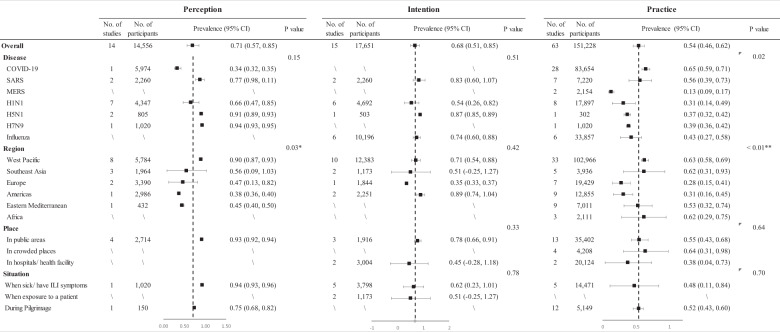
Perception, intention, and practice towards facemask use in subgroups. We performed subgroup analyses of the perception, intention, and practice of facemask use, including different diseases, regions, places, and situations.

In Fig. 3, the meta-analysis showed that 71% of respondents perceived facemasks to be effective for infection prevention, 68% of respondents would wear facemasks, and 54% of respondents wore facemasks for preventing respiratory infections. The subgroup analysis showed that most of the respondents from the West Pacific (90%) perceived facemask use as a good way to prevent the transmission of respiratory infections, while a lower rate of respondents reported the same perception in Southeast Asia (56%), Europe (47%), Eastern Mediterranean (45%), and Americas (38%) (Fig. S3, S4). However, the subgroup analysis showed no significant difference in the rates of intention of wearing facemask in different regions (Fig. S5). There were also no significant differences of the perception and intention of facemask use among different diseases (Fig. S6, S7).

In terms of practice, there was a significant difference of practice rates among different diseases (*p* = 0.02). About 65% of the respondents reported wearing facemasks during the COVID-19 outbreak, 56% of the respondents reported wearing facemasks during the SARS outbreak, and less than 45% reported wearing facemasks during the MERS, H1N1, H5N1, H7N9 and seasonal influenza outbreak (Fig. S8).

**Fig. 4:**
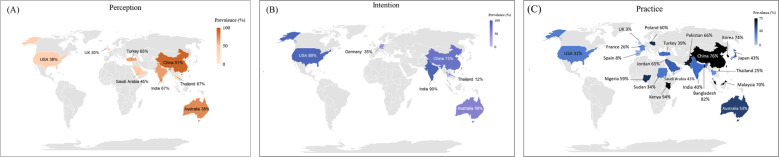
Global prevalence of perception, intention, and practice of facemask use among the general population. **A** Perceived efficacy of facemask for preventing respiratory infection. **B** Intention of facemask use for preventing respiratory infection. **C** Practice of facemask use for preventing respiratory infection during the COVID-19.

The subgroup analysis also showed significant differences in the rates of practice of wearing facemask in different regions (*p* < 0.01). For all of the diseases, the participants reported higher rate of facemask-wearing in the West Pacific (63%), followed by Southeast Asia (62%), Africa (62%), the Eastern Mediterranean (53%), Americas (31%) and Europe (28%) (Fig. S9). During the COVID-19 pandemic, the participants reported the highest rate of facemask-wearing in the West Pacific (83%), followed by Southeast Asia (82%), Eastern Mediterranean (73%) and Africa (62%), Europe (33%), and Americas (32%) (Fig. S10). Global prevalence of perception, intention, and practice of facemask use among different countries or regions are shown in Fig. 4.

The subgroup analysis showed that most of the respondents perceived facemask use in public areas (93%), when sick/having ILI symptoms (94%) or during Pilgrimage (75%) to be effective for preventing transmission (Fig. 3). A smaller proportion of respondents would wear facemasks in public areas (78%), in hospital/health facility (45%), when sick/having ILI symptoms (62%) or after exposure to infected patient (51%) (Fig. S11 and S12). Moreover, 55% of respondents reported to wear facemasks in public areas, 64% reported to wear facemasks in crowded places, 38% reported to wear facemasks in hospital/health facility, 48% reported to wear facemasks when sick/ having ILI symptoms and 52% reported to wear facemasks during Pilgrimage (Fig. S13, S14). There were no significant differences of the perception, intention, and practice of facemask use among different places or situations (Fig. 3). The subgroup analysis of practice by sex and age did not show any significant difference.

### Publication bias, quality assessment, and sensitivity analysis

Significant publication bias was found for the perception (*p* = 0.001) towards facemask use. No publication bias was found for the facemask efficacy, intention, and practice of facemasks use (Fig. S15, *p* > 0.05). The quality assessment was reported in Table S3 for RCTs and Table S4 for studies on perception, intention, and practice of facemask use. The sensitivity analysis showed no significant impact on the RCT outcomes (Fig. S16).

## Discussion

In this systematic review and meta-analysis, we summarized the current evidence on the efficacy of facemask use for the prevention of respiratory infections among the general population. The meta-analysis shows that facemask use can reduce the risk of clinical symptoms of respiratory infection. Moreover, the results of this study showed that the protective effect was more pronounced when the duration of facemask use was longer than two weeks. We also found that 71% of respondents perceived facemask use to be effective for infection prevention, 68% of respondents would wear facemasks, and 54% of respondents wore facemasks for preventing respiratory infections. However, the perception and practice towards facemask use among general population varied in different regions and for different infectious diseases. Our results highlight the importance of facemask use among general populations and provide evidence for the governments and relevant organizations to make efforts to reduce the barriers in the use of facemasks to control the pandemic.

### Efficacy of facemask use

Our results suggest facemask use may significantly reduce the clinical symptoms of respiratory infection. Facemasks are recommended for the prevention of infectious diseases transmitted through droplets and respirators for respiratory aerosols [97]. However, most of the RCTs included in this meta-analysis did not show a statistically significant effect of facemask use for preventing infection in community settings. This might be due to the relatively small sample size and low infection rate in community settings. After the pooled analysis with a much larger sample size, the prevention effect of facemask on infections could be more significant. According to a recent meta-analysis which pooled case-control, retrospective studies and RCTs (published in medRxiv preprint), a protective effect of facemasks was found among non-healthcare workers [15]. However, another previous meta-analysis of RCTs on facemask use reported no significant reduction in laboratory-confirmed influenza infections [14]. The authors of the meta-analysis suggest that the result may be related to limited sample size, only laboratory samples, and suboptimal adherence of facemask use in some studies [14].

A recent study demonstrated that disposable surgical masks could reduce the detection of influenza virus RNA in respiratory droplets and coronavirus RNA in aerosols, and had a higher tendency to reduce coronavirus RNA in respiratory droplets [98]. Evidence from a modeling study also supported the efficacy of facemask, which suggested that broad adoption of facemasks could meaningfully curtail community transmission of COVID-19 and reduce the peak of hospitalizations and deaths [99]. Recently a systematic review suggests that the efficacy of cloth facemask depends on its fabric material and polyester has the best filtration efficiency [100]. Similar with previous studies, our present study also strongly supports that facemask use (eg, surgical masks, longer than 2 weeks) can be an effective and accessible protection of infections of COVID-19 for general population. Further studies need to be conducted to explore the long-term efficacy of various facemasks on prevention of infections.

**Fig. 5:**
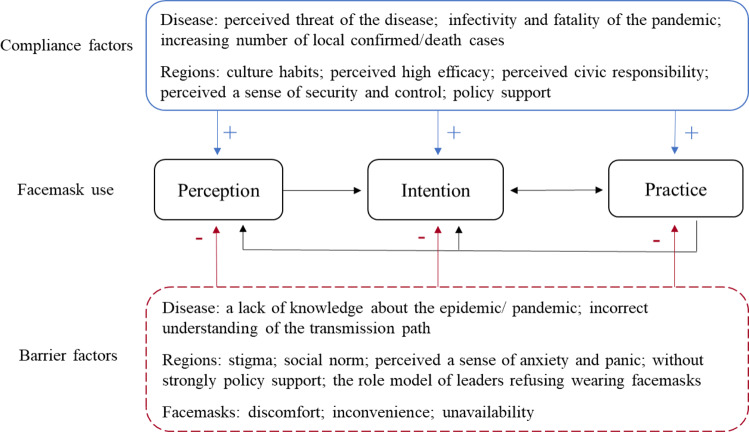
The compliance related factors and barriers in the use of facemask. Factors associated with compliance of facemask use can be divided into threat of disease (e.g. perceived threat of disease) and geographic variation (e.g. perceived civic responsibility). Barrier factors of facemask use can be divided into knowledge of the diseases (e.g. a lack of knowledge of the disease), geographic variation (e.g. stigma), and feeling of facemask use (e.g. discomfort).

Our study also indicates the changing trends of overall perception, intention, and practice of facemask use. The results of our study showed that more than 70% of respondents perceived efficacy of facemask use and 68% of respondents would wear facemasks, but less than half of the respondents had put into practice. The rates of perception of efficacy and practice of facemask use were higher in West Pacific than that in other regions. The higher rates of practice of facemask use in West Pacific might be related to higher perception of efficacy of facemask use. [20] And lower rates of practice of facemask use in Europe and the Americas might be linked to lower perception of efficacy of facemask use. The results of this study also show a gap between intention and practice of facemask use in the Americas. In order to promote the prevention effect of facemasks in community settings, it is important to understand the factors related to compliance and barriers of facemask use (Fig. 5).

### Factors associated with compliance of facemask use

#### Threat of disease

The subgroup analysis showed that different type of disease might affect the practice of facemask use. About 76% of the respondents wore facemasks during the COVID-19 outbreak, 56% of the respondents wore facemasks during the SARS outbreak, and less than 45% of the respondents wore facemasks during the MERS, H1N1, H5N1, H7N9 and seasonal influenza outbreaks. The results of this study indicate that infectivity and fatality of disease may be important factors influencing facemask use. Previous studies reported that perceived fatality of SARS and H1N1 was related to higher practice of facemask use in public areas [38] and crowded places [101], while the respondents who perceived less personal threat of SARS infection would not wear facemasks [54]. The respondents who thought that pandemic influenza was very likely to occur reported higher willingness to comply with wearing facemasks [65]. During the H1N1 outbreak, the most mentioned factors affecting respondents’ decision to use facemask in public areas included the increasing number of local confirmed H1N1 cases and reported deaths of local H1N1 patients [19]. In all, perceived threat of disease could be one of the crucial reasons for the compliance of facemask use. During the COVID-19 pandemic, especially in the early stage, underestimating the infectivity and fatality of the pandemic may have reduced individuals’ vigilance to prevent the COVID-19 pandemic (eg, many individuals in some countries, especially those who did not realize the severity of the COVID-19 pandemic). This is also very common among the young people, who got the information mainly from social media rather than the official government site for COVID-19. [102] The misleading information from the media and some leaders can also hinder the public to accept and use facemasks.

#### Geographic variation

Another influencing factor of facemask use might be different countries and regions. In this study, most of the respondents from the West Pacific perceived that wearing facemasks was beneficial to prevent the transmission of respiratory infections. The practice of facemask use also showed a tendency of higher rates in the West Pacific than in other regions. During the Manchurian plague epidemic in China in 1910, WU Liande, a Chinese doctor, transformed the cotton masks into protective equipment, which was the original “anti-plague mask” [103, 104]. Over the next 100 years, this kind of facemask and its various modified versions were widely used in China. Especially during the SARS outbreak, facemask use was regarded as a remarkable social and health protective behavior and widely recognized by the Hong Kong population. [105] The use of facemask in public has been perceived as a new social norm, a form of civic responsibility, symbolic support for health care providers, and a tool for achieving a sense of control and security in China [105]. During the COVID-19 pandemic, high frequency of facemask use was significantly associated with lower anxiety and depression in China. [106] Regarded as the ‘safety blanket’, facemask use has been widespread in daily life in other Asian countries (eg, Japan), which is more likely to be driven by symbolic dimensions than by scientific evidence alone [107]. High rates of facemask use in East Asian countries may partly reflect the impact of Asian culture (eg, beliefs on facemask) and previous experience on facemask use during the pandemic.

The variation of different rates of facemask use underlines the collective and individual experience about benefits of facemask use, which leads to a high perception of efficacy of facemasks and high practice of facemask use. Besides, the policies supporting facemask use in these regions also have an important effect on the practice of facemask use during the COVID-19 pandemic. Due to the high acceptance of facemask use, the general population in these regions may own a strong motivation to follow these policies and guidelines of wearing facemasks in public areas to fight against COVID-19 pandemic. However, general population in some countries have different beliefs on the benefits of facemask use on curbing respiratory infections, different associations with covering the face and no long-standing habit of wearing facemasks. In the post-SARS era, facemasks have also been associated with stigma, as a sign of negative attributes and perceived to hinder recovery. [105] Besides, facemask-wearing was linked to plague and illness in some European countries, which could produce anxiety and panic in general population. This may partly explain the different individual coping behaviors and community practices among general population in different countries during the COVID-19 pandemic.

### Barriers in facemask use

#### Stigma

The results of this meta-analysis showed that fewer than half of the European respondents perceived the benefits of facemask use in preventing infection and only about one-third of the respondents had worn facemasks during an epidemic/pandemic. The reason of negative attitudes towards facemask use in these countries may partly result from the stigma associated with wearing facemask. In some contexts, masks are implicitly or explicitly opposed to the concepts of transparency and authenticity [108]. Facemask-wearing may thus regarded as a symbol of compliance, regulation, and manipulation, and the government’s opposition to freedom of speech [108]. In an Australian survey, the most perceived barriers to wear a facemask was the presence of stigma [109]. Stigma and prejudice can hinder the intention of facemask use and can potentially cause the feeling of embarrassed or ashamed to wear facemasks [38, 110].

#### Discomfort and inconvenience

Discomfort and inconvenience are commonly reported factors that reduce the compliance of facemask use [38, 109]. Wearing a facemask could cause breathing discomfort, even feeling of suffocation [61]. Other problems reported frequently were humidity, warmth, ear pain, poor fit in size and makeup coming off [110]. Moreover, wearing facemasks might also have a negative impact on interpersonal communication, limiting the making and reading of facial expressions. These negative feelings could impede the process from the intention to practice and reduce the facemasks use duration.

#### Unavailability

Due to the global shortage of medical and disposable surgical facemasks, the availability of facemasks had been quite a problem for general population. With widely spreading COVID-19, demand for personal protective equipment was much higher than average, leading to a worldwide shortage of medical masks for the general population. [111] Learning from the original experience of the Manchurian plague epidemic, the cloth facemasks might be a choice to substitute for medical masks. Despite the lack of high-quality evidence, a study suggested that homemade cloth facemasks showed only 15% less effective than surgical masks in preventing particulate emissions and five times more effective than not wearing facemasks [112]. And it can also be an expression to reduce the stigma of facemask use and build new social norms about facemask use.

There were a few potential limitations in this study. First of all, this study used self-reported clinical symptoms as the outcomes, which could be biased. However, previous RCTs did not test the laboratory-confirmed outcomes for all the subjects, and therefore were unable to reflect the overall infection rate in intervention and control arms. Future RCTs ought to cover all subjects with laboratory-confirmed infection to provide more convincing evidence. Moreover, this study might not be able to include (1) recent ongoing research on facemasks, (2) high-quality research outcomes that might not be published in the studied databases but as technical reports or in gray literature, and (3) other important scientific findings which might not be publishing in English. Last, most of the included studies were observational studies and therefore more RCTs and long-term prospective studies should be needed to confirm the results in the future.

## Conclusion

Overall, this systemic review suggests that facemask use may reduce the respiratory infection in general population in community settings. Given the efficacy of facemask use as a strategy of respiratory infection control, the policy makers should encourage facemask use among the general population for health protection. The present review also shows that there are differences in perception, intention, and practice of facemask use in different regions, and it likely reflects different impacts from various infectious diseases, regional culture and local policies. The governments and related organizations should make effort to increase the compliance of facemask use and reduce barriers associated with the use of facemasks, such as reducing stigma and prejudice on facemask use, public education through media and other communication channels.

## Supplementary information

Supplementary

aj-checklist
