# Supplementary material for: Efficacy and practice of facemask use in general population: a systematic review and meta-analysis
Source: Transl Psychiatry. 2022 Feb 1;12:49. doi: 10.1038/s41398-022-01814-3 (PMC8804079; doi:10.1038/s41398-022-01814-3)
Supplement: Supplementary file 1 — Supplementary [file 41398_2022_1814_MOESM1_ESM.docx]

Supplementary appendix

| Appendix 1: | Search strategy | p 1-2 |
| --- | --- | --- |
| Table S1: | PRISMA checklist | p 3-4 |
| Table S2: | Studies excluded from systematic review | p 5-6 |
| Table S3: | Quality assessment of RCTs | p 7 |
| Table S4: | Quality assessment of studies on perception, intention and practice of facemask use | p 8-9 |
| Figure S1: | Forest plots of RCTs using the facemask for contacts or index patients | p 10 |
| Figure S2: | Forest plots of RCTs conducted in the residence halls, tents or households | p 11 |
| Figure S3: | Forest plot of the difference on the perceived efficacy of facemask use in different WHO regions | p 12 |
| Figure S4: | Forest plot of the difference on the perceived efficacy of facemask use in different countries/regions | p 13 |
| Figure S5: | Forest plot of the difference on the intention of facemask use in different WHO regions | p 14 |
| Figure S6: | Forest plot of the difference on the perceived efficacy of facemask use among different diseases | p 15 |
| Figure S7: | Forest plot of the difference on the intention of facemask use during different diseases outbreak | p 16 |
| Figure S8: | Forest plot of the difference on the practice of facemask use in different diseases outbreak | p 17 |
| Figure S9: | Forest plot of the difference on the practice of facemask use in different WHO regions | p 18 |
| Figure S10: | Forest plot of the difference on the practice of facemask use in different WHO regions during the COVID-19 pandemic | p 19 |
| Figure S11: | Forest plot of the difference on the intention of facemask use in different places | p 20 |
| Figure S12: | Forest plot of the difference on the intention of facemask use under different situations | p 21 |
| Figure S13: | Forest plot of the difference on the practice of facemask use in different places | p 22 |
| Figure S14: | Forest plot of the difference on the practice of facemask use under different situations | p 23 |
| Figure S15: | Funnel plots for studies about facemasks efficacy (A) and perception, intention and practice towards facemasks use (B, C and D) | p 24 |
| Figure S16: | Sensitivity analysis of the included RCTs | p 25 |

**Appendix 1: Search strategy**

We searched PubMed, MEDLINE, Web of Science, Cochrane, medRxiv and bioRxiv database on August 17, 2020, with no language restrictions.

PubMed:

(Influenza A Virus, H5N1 Subtype[MeSH Terms]) OR (H5N1[Title/Abstract]) OR ("influenza A virus"[Title/Abstract]) OR (“Influenza Pandemic”[Title/Abstract]) OR (“Influenza Epidemic”[Title/Abstract]) OR (“Middle East Respiratory Syndrome Coronavirus”[MeSH Terms]) OR (MERS[Title/Abstract]) OR (“Severe Acute Respiratory Syndrome”[MeSH Terms]) OR (“Severe Acute Respiratory Syndrome”[Title/Abstract]) OR (SARS[Title/Abstract]) OR (H1N1[Title/Abstract]) OR (infection*[MeSH Terms]) OR (infection*[Title/Abstract]) OR (influenza[Title/Abstract]) OR (COVID-19[MeSH Terms]) OR (COVID-19[Title/Abstract]) OR (2019-nCoV[Title/Abstract]) OR (“2019 novel coronavirus infection”[Title/Abstract]) AND (mask*[MeSH Terms]) OR (mask[Title/Abstract]) OR (facemask*[Title/Abstract]) OR (“face mask”[Title/Abstract]) OR (“medical mask”[Title/Abstract]) OR (“surgical mask”[Title/Abstract]) OR (“surgical facemask”[Title/Abstract]) OR (“surgical face mask”[Title/Abstract]) OR (N95[Title/Abstract]) OR (respirators[MeSH Terms]) OR (respirators[Title/Abstract])

MEDLINE/ Web of Science (Core)

| No. | Query |
| --- | --- |
| #1 | Ts=influenza A virus, H5N1 subtype |
| #2 | Ts=H5N1 |
| #3 | Ts=influenza A virus |
| #4 | Ts=influenza pandemic |
| #5 | Ts=influenza epidemic |
| #6 | Ts=Middle East Respiratory Syndrome Coronavirus |
| #7 | Ts=MERS |
| #8 | Ts=Severe Acute Respiratory Syndrome |
| #9 | Ts=SARS |
| #10 | Ts=H1N1 |
| #11 | Ts=infection* |
| #12 | Ts=influenza |
| #13 | Ts=COVID-19 |
| #14 | Ts=2019-nCov |
| #15 | Ts=2019 novel coronavirus infection |
| #16 | #1 OR #2 OR #3 OR #4 OR #5 OR #6 OR #7 OR #8 OR #9 OR #10 OR #11 OR #12 OR #13 OR #14 OR #15 |
| #17 | Ts=mask* |
| #18 | Ts=mask |
| #19 | Ts=facemask* |
| #20 | Ts=facemask |
| #21 | Ts=medical mask |
| #22 | Ts=surgical mask |
| #23 | Ts= surgical facemask |
| #24 | Ts= surgical face mask |
| #25 | Ts=N95 |
| #26 | Ts=respirators |
| #27 | #17 OR #18 OR #19 OR #20 OR #21 OR #22 OR #23 OR #24 OR #25 OR #26 |
| #28 | #16 AND #27 |

Cochrane

| No. | Query |
| --- | --- |
| #1 | MeSH descriptor: [influenza A virus, H5N1 subtype] explode all trees |
| #2 | MeSH descriptor: [influenza A virus] explode all trees |
| #3 | MeSH descriptor: [influenza pandemic, 1918-1919] explode all trees |
| #4 | MeSH descriptor: [Middle East Respiratory Syndrome Coronavirus] explode all trees |
| #5 | MeSH descriptor: [Severe Acute Respiratory Syndrome] explode all trees |
| #6 | MeSH descriptor: [influenza A virus, H1N1 subtype] explode all trees |
| #7 | MeSH descriptor: [infections] explode all trees |
| #8 | MeSH descriptor: [influenza, Human] explode all trees |
| #9 | MeSH descriptor: [Coronavirus] explode all trees |
| #10 | influenza A virus, H5N1 subtype OR H5N1 OR influenza A virus OR influenza pandemic OR influenza epidemic OR Middle East Respiratory Syndrome Coronavirus OR MERS OR Severe Acute Respiratory Syndrome OR SARS OR H1N1 OR infection* OR influenza OR COVID-19 OR 2019-nCov OR 2019 novel coronavirus infection |
| #11 | #1 OR #2 OR #3 OR #4 OR #5 OR #6 OR #7 OR #8 OR #9 OR #10 |
| #12 | MeSH descriptor: [Masks] explode all trees |
| #13 | Mask* OR mask OR facemask* OR face mask OR medical mask OR surgical mask OR surgical facemask OR surgical face mask OR N95 OR respirators |
| #14 | #12 OR #13 |
| #15 | #11 AND #14 |

**Table S1. PRISMA checklist**

| **Section/ topic** | **#** | **Checklist item** | **Reported on page #** |
| --- | --- | --- | --- |
| **TITLE** | | |  |
| Title | 1 | Identify the report as a systematic review, meta-analysis, or both. | p 1 |
| **ABSTRACT** | | |  |
| Structured summary | 2 | Provide a structured summary including, as applicable: background; objectives; data sources; study eligibility criteria, participants, and interventions; study appraisal and synthesis methods; results; limitations; conclusions and implications of key findings; systematic review registration number. | p 3 |
| **INTRODUCTION** | | |  |
| Rationale | 3 | Describe the rationale for the review in the context of what is already known. | p 4-5 |
| Objectives | 4 | Provide an explicit statement of questions being addressed with reference to participants, interventions, comparisons, outcomes, and study design (PICOS). | p 5 |
| **METHODS** | | |  |
| Protocol and registration | 5 | Indicate if a review protocol exists, if and where it can be accessed (e.g., Web address), and, if available, provide registration information including registration number. | P6 |
| Eligibility criteria | 6 | Specify study characteristics (e.g., PICOS, length of follow-up) and report characteristics (e.g., years considered, language, publication status) used as criteria for eligibility, giving rationale. | p 6 |
| Information sources | 7 | Describe all information sources (e.g., databases with dates of coverage, contact with study authors to identify additional studies) in the search and date last searched. | p 6 |
| Search | 8 | Present full electronic search strategy for at least one database, including any limits used, such that it could be repeated. | Appendix |
| Study selection | 9 | State the process for selecting studies (i.e., screening, eligibility, included in systematic review, and, if applicable, included in the meta-analysis). | p 6-7, Fig.1 |
| Data collection process | 10 | Describe method of data extraction from reports (e.g., piloted forms, independently, in duplicate) and any processes for obtaining and confirming data from investigators. | p 6-7 |
| Data items | 11 | List and define all variables for which data were sought (e.g., PICOS, funding sources) and any assumptions and simplifications made. | Appendix |
| Risk of bias in individual studies | 12 | Describe methods used for assessing risk of bias of individual studies (including specification of whether this was done at the study or outcome level), and how this information is to be used in any data synthesis. | p 7-8 |
| Summary measures | 13 | State the principal summary measures (e.g., risk ratio, difference in means). | p 7-8 |
| Synthesis of results | 14 | Describe the methods of handling data and combining results of studies, if done, including measures of consistency (e.g., I^2^) for each meta-analysis. | p 7-8 |
| Risk of bias across studies | 15 | Specify any assessment of risk of bias that may affect the cumulative evidence (e.g., publication bias, selective reporting within studies). | p 8 |
| Additional analyses | 16 | Describe methods of additional analyses (e.g., sensitivity or subgroup analyses, meta-regression), if done, indicating which were pre-specified. | p 8 |
| **RESULTS** | | |  |
| Study selection | 17 | Give numbers of studies screened, assessed for eligibility, and included in the review, with reasons for exclusions at each stage, ideally with a flow diagram. | p 8, Fig. 1 |
| Study characteristics | 18 | For each study, present characteristics for which data were extracted (e.g., study size, PICOS, follow-up period) and provide the citations. | p 8-9, Table 1 |
| Risk of bias within studies | 19 | Present data on risk of bias of each study and, if available, any outcome level assessment (see item 12). | p 11, Appendix |
| Results of individual studies | 20 | For all outcomes considered (benefits or harms), present, for each study: (a) simple summary data for each intervention group (b) effect estimates and confidence intervals, ideally with a forest plot. | p 8-9, Fig. 2 |
| Synthesis of results | 21 | Present results of each meta-analysis done, including confidence intervals and measures of consistency. | p 10-11, Fig. 3 |
| Risk of bias across studies | 22 | Present results of any assessment of risk of bias across studies (see Item 15). | p 15, Appendix |
| Additional analysis | 23 | Give results of additional analyses, if done (e.g., sensitivity or subgroup analyses, meta-regression [see Item 16]). | p13-15, Fig. 2, 3 |
| **DISCUSSION** | | |  |
| Summary of evidence | 24 | Summarize the main findings including the strength of evidence for each main outcome; consider their relevance to key groups (e.g., healthcare providers, users, and policy makers). | p 11-17 |
| Limitations | 25 | Discuss limitations at study and outcome level (e.g., risk of bias), and at review-level (e.g., incomplete retrieval of identified research, reporting bias). | p 17 |
| Conclusions | 26 | Provide a general interpretation of the results in the context of other evidence, and implications for future research. | p 17 |
| **FUNDING** | | |  |
| Funding | 27 | Describe sources of funding for the systematic review and other support (e.g., supply of data); role of funders for the systematic review. | p 18 |

**Table S2. Studies excluded from systematic review**

| **Study** | | **Reason for Exclusion** |
| --- | --- | --- |
| 1. | Chan, D. K.-C., et al. (2015). Preventing the spread of H1N1 influenza infection during a pandemic: autonomy-supportive advice versus controlling instruction. J Behav Med 38(3): 416-426. | Study design does not fit the inclusion criteria |
| 2. | Cheng, C. and C. S. K. Tang (2004). The psychology behind the masks: Psychological responses to the severe acute respiratory syndrome outbreak in different regions. Asian Journal of Social Psychology 7(1): 3-7. | Outcome of interest not measured |
| 3. | Chung, P.-K., et al. (2017). The process by which perceived autonomy support predicts motivation, intention, and behavior for seasonal influenza prevention in Hong Kong older adults. BMC Public Health 18(1): 65. | Outcome of interest not measured |
| 4. | Elachola, H., et al. (2020). COVID-19: Facemask use prevalence in international airports in Asia, Europe and the Americas, March 2020. Travel Med Infect Dis: 101637. | Study design does not fit the inclusion criteria |
| 5. | Ferng, Y.-h., et al. (2011). Barriers to mask wearing for influenza-like illnesses among urban Hispanic households. Public Health Nurs 28(1): 13-23. | Outcome of interest not measured |
| 6. | Ho, H. S. (2012). Use of face masks in a primary care outpatient setting in Hong Kong: Knowledge, attitudes and practices. Public Health 126(12): 1001-1006. | Participants do not fit the  inclusion criteria |
| 7. | Kuo, P. C., et al. (2011). Avian influenza risk perception and preventive behavior among traditional market workers and shoppers in Taiwan: practical implications for prevention. PLoS One 6(9): e24157. | Outcome of interest not measured |
| 8. | Lau, J. T. F., et al. (2004). Probable secondary infections in households of SARS patients in Hong Kong. Emerg Infect Dis 10(2): 235-243. | Study design does not fit the inclusion criteria |
| 9. | Lau, J. T. F., et al. (2004). SARS transmission, risk factors, and prevention in Hong Kong. Emerg Infect Dis 10(4): 587-592. | Study design does not fit the inclusion criteria |
| 10. | Lau, J. T. F., et al. (2011). Changes in knowledge, perceptions, preventive behaviours and psychological responses in the pre-community outbreak phase of the H1N1 epidemic. Epidemiol Infect 139(1): 80-90. | Article reports on an already included study |
| 11. | Li, Y. G. (2011). The secret behind the mask. Indoor Air 21(2): 89-91. | Letters |
| 12. | Lo, J. Y., et al. (2005). Respiratory infections during SARS outbreak, Hong Kong, 2003. Emerg Infect Dis 11(11): 1738-1741. | Outcome of interest not measured |
| 13. | Ma, Q. X., et al. (2020). Potential utilities of mask-wearing and instant hand hygiene for fighting SARS-CoV-2. J Med Virol. | Study design does not fit the inclusion criteria |
| 14. | Ma, X. W., et al. (2014). Knowledge, attitudes and practices relating to influenza A(H7N9) risk among live poultry traders in Guangzhou City, China. BMC Infect Dis 14: 12. | Participants do not fit the  inclusion criteria |
| 15. | Mao, L. (2011). Evaluating the combined effectiveness of influenza control strategies and human preventive behavior. PLoS One 6(10): e24706. | Study design does not fit the inclusion criteria |
| 16. | Morishima, M. and K. Kishida (2018). Understanding attitudes toward hygiene mask use in Japanese daily life by using a repeated cross-sectional survey. Work 61(2): 303-311. | Study design does not fit the inclusion criteria |
| 17. | Perez, V., et al. (2012). Stress, adherence to preventive measures for reducing influenza transmission and influenza-like illness. J Epidemiol Community Health 66(7): 605-610. | Outcome of interest not measured |
| 18. | Roberge, R. (2011). Facemask use by children during infectious disease outbreaks. Biosecur Bioterror 9(3): 225-231. | Outcome of interest not measured |
| 19. | Seale, H., et al. (2012). Examining the knowledge, attitudes and practices of domestic and international university students towards seasonal and pandemic influenza. BMC Public Health 12: 6. | Outcome of interest not measured |
| 20. | Sin, M. S. Y. (2016). Masking fears: SARS and the politics of public health in China. Crit Public Health 26(1): 88-98. | Study design does not fit the inclusion criteria |
| 21. | Skaria, S. D. and G. C. Smaldone (2014). Respiratory source control using surgical masks with nanofiber media. Annals of Occupational Hygiene 58(6): 771-781. | Study design does not fit the inclusion criteria |
| 22. | Syed, Q., et al. (2003). Behind the mask. Journey through an epidemic: some observations of contrasting public health responses to SARS. J Epidemiol Community Health 57(11): 855-856. | Outcome of interest not measured |
| 23. | Takahashi, S., et al. (2017). Public preventive awareness and preventive behaviors during a major influenza epidemic in Fukui, Japan. J Infect Public Health 10(5): 637-643. | Outcome of interest not measured |
| 24. | Tracht, S. M., et al. (2012). Economic analysis of the use of facemasks during pandemic (H1N1) 2009. J Theor Biol 300: 161-172. | Study design does not fit the inclusion criteria |
| 25. | Uchida, M., et al. (2018). High vaccination coverage is associated with low epidemic level of seasonal influenza in elementary schools: an observational study in Matsumoto City, Japan. BMC Infect Dis 18(1): 128. | Outcome of interest not measured |
| 26. | Weiss, M. M., et al. (2007). Disrupting the transmission of influenza: face masks and ultraviolet light as control measures. Am J Public Health 97 Suppl 1: S32-37. | Study design does not fit the inclusion criteria |
| 27. | Zhang, C.-Q., et al. (2019). Health Beliefs of Wearing Facemasks for Influenza A/H1N1 Prevention: A Qualitative Investigation of Hong Kong Older Adults. Asia Pac J Public Health 31(3): 246-256. | Study design does not fit the inclusion criteria |
| 28. | Zhang, L. J., et al. (2013). Protection by Face Masks against Influenza A(H1N1)pdm09 Virus on Trans-Pacific Passenger Aircraft, 2009. Emerg Infect Dis 19(9): 1403-1410. | Study design does not fit the inclusion criteria |
| 29. | Zhao, J.-h., et al. (2009). Personal protective and healthcare seeking behaviors urban residents before and during an influenza pandemic in Beijing. Zhonghua Liu Xing Bing Xue Za Zhi 30(11): 1121-1124. | Outcome of interest not measured |
| 30. | Azman, A. S., et al. (2013). Household transmission of influenza A and B in a school-based study of non-pharmaceutical interventions. Epidemics 5(4): 181-186. | Intervention does not fit the inclusion criteria |
| 31. | Cowling, B. J., et al. (2009). Facemasks and hand hygiene to prevent influenza transmission in households: a cluster randomized trial. Ann Intern Med 151(7): 437-446. | Intervention does not fit the inclusion criteria |
| 32. | Cowling, B. J., et al. (2014). Modes of transmission of influenza B virus in households. PLoS One 9(9): e108850. | Outcome of interest not measured |
| 33. | Emamian, M. H., et al. (2013). Respiratory Tract Infections and its Preventive Measures among Hajj Pilgrims, 2010: A Nested Case Control Study. Int J Prev Med 4(9): 1030-1035. | Study design does not fit the inclusion criteria |
| 34. | Larson, E. L., et al. (2010). Impact of non-pharmaceutical interventions on URIs and influenza in crowded, urban households. Public Health Rep 125(2): 178-191. | Intervention does not fit the inclusion criteria |
| 35. | Lau, M. S. Y., et al. (2015). Inferring influenza dynamics and control in households. Proc Natl Acad Sci U S A 112(29): 9094-9099. | Intervention does not fit the inclusion criteria |
| 36. | Rashid, H., et al. (2012). Unmasking Masks in Makkah: Preventing Influenza at Hajj. Clinical Infectious Diseases 54(1): 151-153. | Study design does not fit the inclusion criteria |
| 37. | Schimit, P. H. T. and L. H. A. Monteiro (2010). Who should wear mask against airborne infections? Altering the contact network for controlling the spread of contagious diseases. Ecological Modelling 221(9): 1329-1332. | Study design does not fit the inclusion criteria |
| 38. | Simmerman, J. M., et al. (2011). Findings from a household randomized controlled trial of hand washing and face masks to reduce influenza transmission in Bangkok, Thailand. Influenza Other Respir Viruses 5(4): 256-267. | Intervention does not fit the inclusion criteria |
| 39. | Suess, T., et al. (2011). Facemasks and intensified hand hygiene in a German household trial during the 2009/2010 influenza A(H1N1) pandemic: adherence and tolerability in children and adults. Epidemiol Infect 139(12): 1895-1901. | Outcome of interest not measured |
| 40. | Li, et al., (2020). Internet use, risk awareness, and demographic characteristics associated with engagement in preventive behaviors and testing: cross-sectional survey on COVID-19 in the United States. J Med Internet Res 22(6). | Outcome of interest not measured |

**Table S3. Quality assessment of RCTs.**

| **RCT** | **Risk of Bias (low, high, unclear)** | | | | | | | |
| --- | --- | --- | --- | --- | --- | --- | --- | --- |
|  | Random Sequence Generation | Allocation Concealment | Performance Bias | Detection Bias | Attrition Bias | Reporting Bias | Other Bias |  |
| Aiello et al., 2010^11^ | Low | Low | High | Low | Low | Low | None |  |
| Aiello et al., 2012^12^ | Low | Low | Low | Low | Low | Low | None |  |
| Barasheed et al., 2014^13^ | High | Low | Low | Low | Low | Low | None |  |
| Canini et al., 2010^7^ | Low | Low | Low | Low | Low | Low | None |  |
| Cowling et al. 2008^6^ | Unclear | Unclear | Low | Low | Low | Low | None |  |
| MacIntyre et al., 2009^9^ | Low | Low | Low | Low | Low | Low | None |  |
| MacIntyre et al., 2016^10^ | Unclear | Low | Low | Low | Low | Low | None |  |
| Suess et al., 2012^8^ | Unclear | Low | Low | Low | Low | Low | None |  |

**Table S4. Quality assessment of studies on perception, intention and practice of facemask use.**

| Study | **Q1** | **Q2** | **Q3** | **Q4** | **Q 5** | **Q 6** | **Q 7** | **Q 8** | **Q 9** | **Q 10** | **Total** |
| --- | --- | --- | --- | --- | --- | --- | --- | --- | --- | --- | --- |
| Abdulah et al., 2020^86^ | 2 | 2 | 2 | 2 | 2 | 1 | 2 | 2 | 2 | 2 | 19 |
| Agüero et al., 2011^75^ | 2 | 2 | 1 | 2 | 2 | 2 | 1 | 2 | 2 | 2 | 18 |
| Ahmad et al., 2017^57^ | 1 | 1 | 1 | 1 | 1 | 1 | 2 | 2 | 1 | 2 | 13 |
| Akan et al., 2010^22^ | 2 | 2 | 1 | 2 | 2 | 1 | 2 | 2 | 2 | 2 | 18 |
| Al-Jasser et al., 2012^76^ | 2 | 2 | 2 | 2 | 2 | 2 | 1 | 2 | 2 | 2 | 19 |
| Allison et al., 2010^58^ | 2 | 1 | 1 | 1 | 2 | 2 | 2 | 2 | 2 | 2 | 17 |
| Al-Mohrej et al., 2016^59^ | 2 | 2 | 1 | 2 | 2 | 1 | 2 | 2 | 2 | 2 | 18 |
| Alqahtani et al., 2016a^60^ | 2 | 2 | 1 | 2 | 2 | 2 | 2 | 2 | 2 | 2 | 19 |
| Alqahtani et al., 2016b^61^ | 2 | 2 | 2 | 2 | 2 | 2 | 2 | 2 | 2 | 2 | 20 |
| Alqahtani et al., 2019^62^ | 2 | 2 | 1 | 2 | 2 | 1 | 2 | 2 | 2 | 2 | 18 |
| Alzoubi et al., 2020^68^ | 2 | 1 | 2 | 2 | 2 | 2 | 2 | 2 | 2 | 2 | 19 |
| Ayhan et al., 2020^87^ | 2 | 1 | 2 | 2 | 2 | 1 | 1 | 2 | 2 | 2 | 17 |
| Azlan et al., 2020^69^ | 2 | 2 | 1 | 2 | 2 | 1 | 2 | 2 | 2 | 2 | 18 |
| Azman et al., 2017^63^ | 2 | 2 | 2 | 2 | 2 | 1 | 1 | 2 | 2 | 2 | 18 |
| Balaban et al., 2012^64^ | 2 | 2 | 2 | 2 | 2 | 2 | 2 | 2 | 2 | 2 | 20 |
| Barr et al., 2008^65^ | 2 | 2 | 1 | 2 | 2 | 2 | 1 | 2 | 2 | 2 | 18 |
| Beckage et al., 2020^94^ | 2 | 2 | 2 | 1 | 1 | 1 | 2 | 2 | 2 | 2 | 17 |
| Bowman et al., 2020^95^ | 2 | 2 | 2 | 2 | 2 | 1 | 2 | 2 | 2 | 2 | 19 |
| CDC., 2004^28^ | 2 | 2 | 1 | 1 | 2 | 2 | 2 | 2 | 2 | 2 | 18 |
| Chan et al., 2015^66^ | 2 | 2 | 2 | 2 | 2 | 2 | 2 | 2 | 2 | 2 | 20 |
| Chaudhary et al., 2010^67^ | 2 | 2 | 1 | 1 | 2 | 2 | 1 | 2 | 2 | 2 | 17 |
| Chen et al., 2020^70^ | 2 | 2 | 2 | 2 | 2 | 2 | 1 | 2 | 2 | 2 | 19 |
| Cheng et al., 2020^96^ | 2 | 2 | 2 | 0 | 1 | 1 | 1 | 1 | 2 | 2 | 14 |
| Chen et al., 2020^88^ | 2 | 2 | 2 | 0 | 2 | 2 | 2 | 2 | 2 | 2 | 18 |
| Chuang et al., 2015^29^ | 2 | 2 | 2 | 2 | 2 | 1 | 2 | 2 | 2 | 2 | 19 |
| Clements et al., 2020^26^ | 2 | 2 | 1 | 2 | 2 | 0 | 0 | 2 | 2 | 2 | 15 |
| Cowling et al., 2020^71^ | 2 | 2 | 1 | 2 | 2 | 2 | 2 | 2 | 2 | 2 | 19 |
| Deris et al., 2010^30^ | 2 | 1 | 2 | 2 | 2 | 1 | 2 | 2 | 2 | 2 | 18 |
| Etingen et al., 2013^31^ | 2 | 1 | 1 | 2 | 2 | 2 | 2 | 2 | 2 | 2 | 18 |
| Ferdous et al., 2020^72^ | 2 | 1 | 2 | 2 | 2 | 1 | 2 | 2 | 2 | 2 | 18 |
| Gautret et al., 2011^32^ | 1 | 1 | 1 | 2 | 2 | 2 | 1 | 2 | 2 | 2 | 16 |
| Geldsetzer et al., 2020^27^ | 2 | 2 | 2 | 2 | 2 | 2 | 0 | 2 | 2 | 2 | 18 |
| Griffiths et al., 2010^33^ | 2 | 1 | 1 | 2 | 2 | 1 | 2 | 2 | 2 | 2 | 17 |
| Gu et al., 2015^34^ | 2 | 2 | 2 | 2 | 2 | 1 | 2 | 2 | 2 | 2 | 19 |
| Gunasekaran et al., 2020^73^ | 2 | 2 | 2 | 2 | 2 | 2 | 2 | 2 | 2 | 2 | 20 |
| Haischer et al., 2020^93^ | 2 | 1 | 1 | 2 | 2 | 1 | 1 | 2 | 2 | 2 | 16 |
| Hashim et al., 2016^35^ | 2 | 2 | 2 | 2 | 2 | 1 | 2 | 2 | 2 | 2 | 19 |
| Hayat et al., 2020^74^ | 2 | 2 | 1 | 2 | 2 | 1 | 2 | 2 | 2 | 2 | 18 |
| Hezima et al., 2020^89^ | 2 | 1 | 2 | 2 | 2 | 1 | 1 | 2 | 2 | 2 | 17 |
| Hickey et al., 2014^23^ | 2 | 2 | 2 | 2 | 2 | 2 | 1 | 2 | 2 | 2 | 19 |
| Huang et al., 2020^77^ | 2 | 1 | 1 | 2 | 2 | 2 | 2 | 2 | 2 | 2 | 18 |

| Continued | **Q1** | **Q2** | **Q3** | **Q4** | **Q 5** | **Q 6** | **Q 7** | **Q 8** | **Q 9** | **Q 10** | **Total** |
| --- | --- | --- | --- | --- | --- | --- | --- | --- | --- | --- | --- |
| Ikpama et al., 2020^25^ | 2 | 1 | 1 | 2 | 2 | 1 | 1 | 2 | 2 | 2 | 19 |
| Jang et al., 2019^36^ | 2 | 2 | 2 | 2 | 2 | 1 | 2 | 2 | 2 | 2 | 19 |
| Kamate et al., 2010^37^ | 2 | 1 | 2 | 2 | 2 | 1 | 1 | 2 | 2 | 2 | 17 |
| Kantor et al., 2020^90^ | 2 | 2 | 2 | 2 | 2 | 1 | 2 | 2 | 2 | 2 | 19 |
| Lau et al., 2004^38^ | 2 | 2 | 1 | 2 | 2 | 2 | 2 | 2 | 2 | 2 | 19 |
| Lau et al., 2004^39^ | 2 | 1 | 1 | 2 | 2 | 2 | 2 | 2 | 2 | 2 | 18 |
| Lau et al., 2005a^18^ | 2 | 2 | 1 | 2 | 2 | 2 | 2 | 2 | 2 | 2 | 19 |
| Lau et al., 2005b^40^ | 2 | 1 | 1 | 2 | 2 | 2 | 2 | 2 | 2 | 2 | 18 |
| Lau et al., 2007^41^ | 2 | 1 | 2 | 2 | 2 | 2 | 2 | 2 | 2 | 2 | 19 |
| Lau et al., 2008^42^ | 2 | 1 | 1 | 2 | 2 | 2 | 2 | 2 | 2 | 2 | 18 |
| Lau et al., 2009^19^ | 2 | 1 | 1 | 2 | 2 | 2 | 2 | 2 | 2 | 2 | 18 |
| Lau et al., 2010^43^ | 2 | 1 | 2 | 2 | 2 | 2 | 2 | 2 | 2 | 2 | 19 |
| Lee et al., 2020^79^ | 2 | 2 | 1 | 2 | 2 | 1 | 2 | 2 | 2 | 2 | 18 |
| Liu et al., 2020^80^ | 2 | 1 | 1 | 2 | 2 | 2 | 2 | 2 | 2 | 2 | 18 |
| Matusiak et al., 2020^91^ | 2 | 1 | 1 | 2 | 2 | 1 | 1 | 2 | 2 | 2 | 16 |
| Meilicke et al., 2013^44^ | 2 | 2 | 2 | 2 | 2 | 2 | 2 | 2 | 2 | 2 | 20 |
| Memish et al., 2012^45^ | 2 | 2 | 1 | 2 | 2 | 2 | 1 | 2 | 2 | 2 | 18 |
| Mo et al., 2015^46^ | 2 | 2 | 1 | 2 | 2 | 2 | 2 | 2 | 2 | 2 | 19 |
| Mohammed et al., 2019^47^ | 2 | 1 | 1 | 2 | 2 | 1 | 1 | 2 | 2 | 2 | 16 |
| Quaife et al., 2020^81^ | 2 | 1 | 0 | 1 | 2 | 1 | 2 | 2 | 2 | 2 | 15 |
| Rahman & Sathi, 2020^92^ | 2 | 1 | 2 | 2 | 2 | 1 | 1 | 2 | 2 | 2 | 17 |
| Szepietowski et al., 2020^82^ | 2 | 1 | 1 | 2 | 2 | 1 | 1 | 2 | 2 | 2 | 16 |
| Tang et al., 2003^48^ | 1 | 1 | 1 | 1 | 1 | 2 | 0 | 2 | 2 | 1 | 12 |
| Tang et al., 2004^78^ | 2 | 2 | 1 | 2 | 2 | 2 | 0 | 2 | 2 | 2 | 17 |
| Taylor et al., 2009^49^ | 2 | 2 | 1 | 2 | 2 | 2 | 1 | 2 | 2 | 2 | 18 |
| Taylor et al., 2012^50^ | 2 | 2 | 1 | 1 | 2 | 2 | 2 | 2 | 2 | 2 | 18 |
| Tobaiqy et al., 2020^83^ | 2 | 2 | 2 | 2 | 2 | 2 | 2 | 2 | 2 | 2 | 20 |
| Uchida et al., 2017^51^ | 2 | 1 | 1 | 2 | 2 | 1 | 2 | 2 | 2 | 2 | 17 |
| Van Cauteren et al., 2012^52^ | 2 | 2 | 2 | 2 | 2 | 2 | 2 | 2 | 2 | 2 | 20 |
| Wada et al., 2012^53^ | 2 | 2 | 1 | 2 | 2 | 1 | 2 | 2 | 2 | 2 | 18 |
| Wadood et al., 2020^84^ | 2 | 1 | 2 | 2 | 2 | 2 | 2 | 2 | 2 | 2 | 19 |
| Wong et al., 2005^54^ | 2 | 2 | 1 | 2 | 2 | 2 | 1 | 2 | 2 | 2 | 18 |
| Wu et al., 2016^55^ | 2 | 2 | 1 | 2 | 2 | 1 | 2 | 2 | 2 | 2 | 18 |
| Xu et al., 2020^85^ | 2 | 1 | 2 | 2 | 2 | 1 | 2 | 2 | 2 | 2 | 18 |
| Zhang et al., 2016^56^ | 2 | 1 | 1 | 2 | 2 | 2 | 2 | 2 | 2 | 2 | 18 |
| Zhong et al., 2020^24^ | 2 | 2 | 1 | 2 | 2 | 1 | 2 | 2 | 2 | 2 | 18 |

**Figure S1. Forest plots of RCTs using the facemask for contacts or index patients.**

**Figure S2. Forest plots of RCTs conducted in the residence halls, tents or households.**

**Figure S3. Forest plot of the difference on the perceived efficacy of facemask use in different WHO regions.**

**Figure S4. Forest plot of the difference on the perceived efficacy of facemask use in different countries/regions.**

**Figure S5. Forest plot of the difference on the intention of facemask use in different WHO regions.**

**Figure S6. Forest plot of the difference on the perceived efficacy of facemask use among different diseases.**

**Figure S7. Forest plot of the difference on the intention of facemask use during different diseases outbreak.**

**
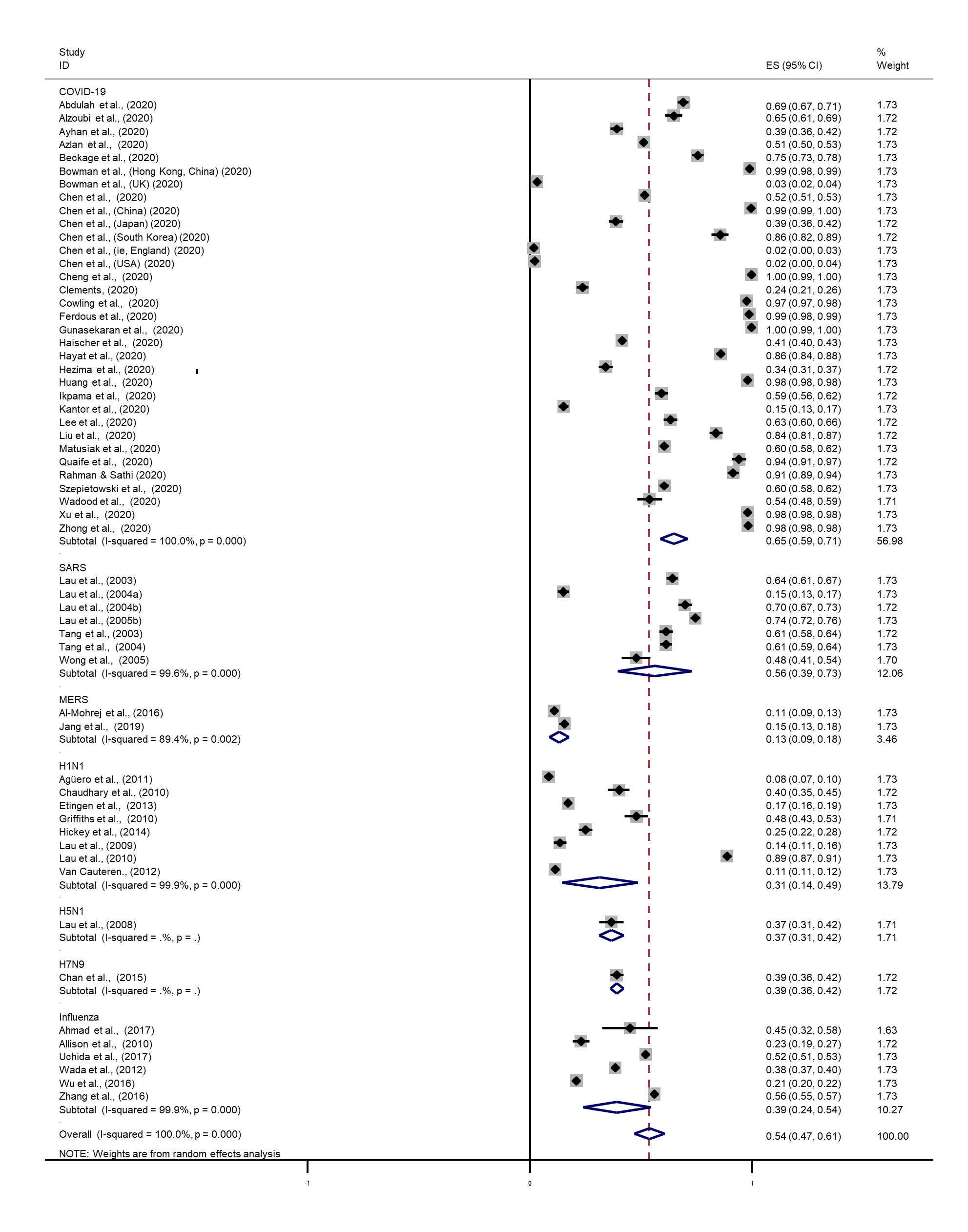
**

**Figure S8. Forest plot of the difference on the practice of facemask use in different diseases outbreak.**

**
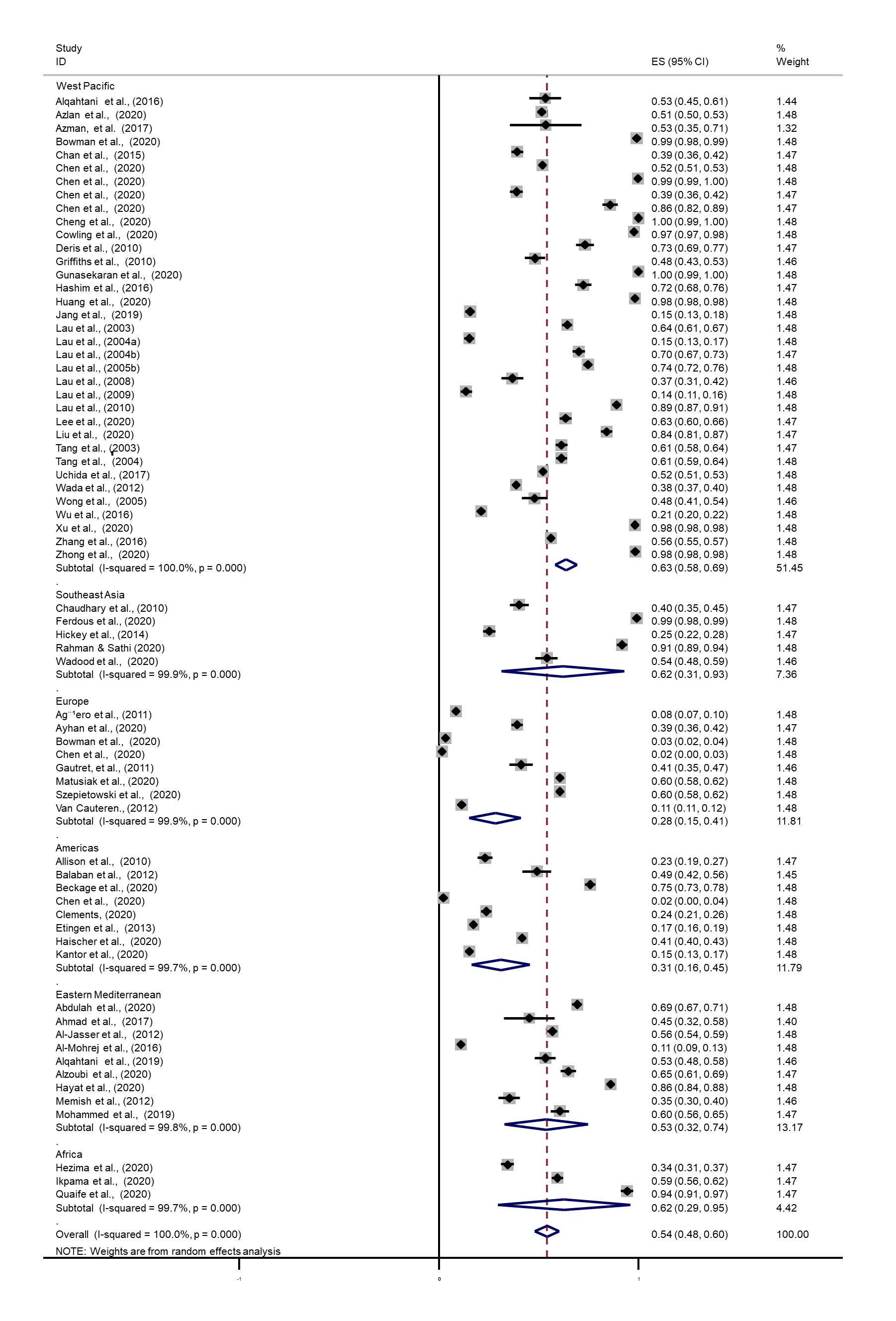
**

**Figure S9. Forest plot of the difference on the practice of facemask use in different WHO regions.**

**Figure S10. Forest plot of the difference on the practice of facemask use in different WHO regions during the COVID-19 pandemic.**

**Figure S11. Forest plot of the difference on the intention of facemask use in different places.**

**Figure S12. Forest plot of the difference on the intention of facemask use under different situations.**

**Figure S13. Forest plot of the difference on the practice of facemask use in different places.**

**Figure S14. Forest plot of the difference on the practice of facemask use under different situations.**


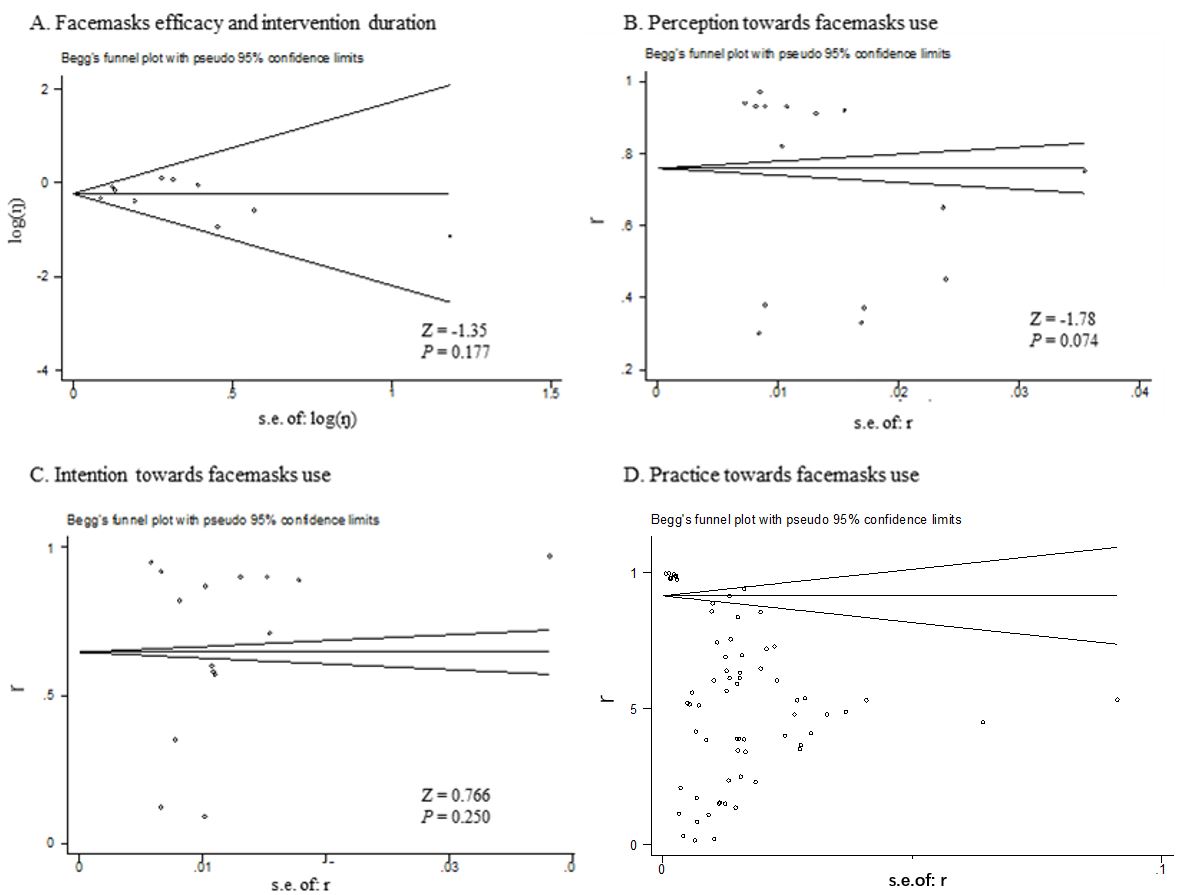


**Figure S15. Funnel plots for studies about facemasks efficacy (A) and perception, intention and practice towards facemasks use (B, C and D).**

**Figure S16. Sensitivity analysis of the included RCTs.**
